# Effect of Blastocyst Morphology and Developmental Rate on Euploidy and Live Birth Rates in PGT-A Cycles: A Retrospective Cohort Study

**DOI:** 10.1007/s43032-025-01818-6

**Published:** 2025-02-21

**Authors:** Xiaojiao Chen, Yong Fan, Hui Ji, Lin Zhou, Xun Wu, Yi Wei, Shanren Cao, Junqiang Zhang, Xiufeng Ling

**Affiliations:** Department of Reproductive Medicine, Women’S Hospital of Nanjing Medical University, Nanjing Women and Children’S Healthcare Hospital, 123 Tianfeixiang, Mochou Road, Nanjing, 210004 Jiangsu China

**Keywords:** PGT-A, Blastocyst morphology, Developmental rate, Euploidy rate, Live birth rate

## Abstract

This study was to evaluate the relationship between morphology or developmental rate of blastocysts and ploidy status in preimplantation genetic testing for aneuploidy (PGT-A) cycles, or live birth rates in single euploid frozen-thawed embryo transfer (FET) cycles. We focused on infertile patients who underwent PGT-A procedures, following with single euploid FET cycles in the assisted reproduction center from January 2016 to December 2022. Blastocysts were categorized for biopsy, and euploid embryos would be selected for transfer. Multivariate logistic regression was used to assess the effects of blastocoel expansion degree, inner cell mass (ICM) and trophectoderm (TE) grades and developmental stage (Day 5, 6 and 7) on euploidy and live birth rates. A total of 4172 blastocysts from 941 PGT-A cycles were included. Expansion 4 were associated with lower euploidy rate than expansion 5 (*P* = 0.011) and 6 (*P* = 0.001). Better ICM (*P* < 0.05 for A compared to B grade) increased blastocyst euploidy. Euploidy rate was significantly associated with A grade TE (*P* < 0.001). However, no relationship existed between blastocyst euploidy and developmental rate. Furthermore, live birth rates had no significant effect on most of morphological parameters in euploidy blastocyst FET cycles, only A grade TE was shown higher live birth rate than C grade (*P* = 0.024). The rate of euploidy in morphologically poor blastocysts is low in the cohort, but the developmental rate does not associate with euploidy. Moreover, only TE grades take association with live birth rates, when the single euploidy blastocyst was transplanted.

## Introduction

Accurate assessment of embryo quality to improve the rate of live birth is an important development direction in the field of reproductive medicine. Reproductive centers usually use the traditional morphological assessment method, such as the Gardner and Schoolcraft grading system, the Society for Assisted Reproductive Technology (SART), and the Istanbul consensus workshop, to evaluate the quality of embryos [1–3], mainly including the degree of expansion, and the appearances of inner cell mass (ICM) and trophectoderm (TE) in terms of the blastocyst stage. As a routine non-invasive evaluation system, the effectiveness of screening high-quality blastocysts based on their appearance has been confirmed [4–6], but the morphokinetics of embryo are susceptible to the external culture environment, and the judgment of embryo quality is often disturbed by subjective experience [7, 8].

On the other hand, preimplantation genetic testing of aneuploidy (PGT-A) has developed rapidly with the advancement of technology, which detects the euploidy state of blastocysts through TE cell biopsy and sequencing. Although PGT-A reflects the chromosomal status of blastocysts, its clinical effectiveness on the rate of live birth remains controversy [9, 10]. Considering the damage caused by the biopsy process and the interference of chimeric embryos with the diagnosis, PGT-A is strictly limited to some cases of infertility, such as advanced maternal age, recurrent implantation failure or pregnancy loss, and severe male factor [11].

The purpose of morphological screening and PGT-A is to determine the best blastocysts for transfer to improve the success rate of assisted reproductive treatment. In PGT-A cycles, blastocysts are firstly graded according to their morphological characteristics before biopsy, and morphological parameters, as the necessary indicators, determine the order of transfer in subsequent single euploid frozen-thawed embryo transfer (FET) cycles [12]. Some studies have shown a close association between blastocyst morphology and euploidy [13–15], but a weak correlation has also been reported [16]. A consecutive case series study found that more than half of blastocysts with good morphological grade were later identified as being aneuploid [17]. Additionally, the effect of developmental rate on blastocyst euploidy is also controversial [18, 19]. Thus, it is still unknown whether conventional parameters of morphology and developmental rate correlate with the chromosomal constitution of blastocysts.

In view of the above contradictory conclusions, more information and analysis on the association between blastocyst characteristics and euploidy rate may help to screen high-quality embryos and improve clinical outcomes. Our study was to investigate whether the morphology and developmental rate of blastocyst correlate with embryo euploidy status in PGT-A cycles. Furthermore, the association between these conventional parameters and live birth rate was also evaluated in the following single euploid FET cycles.

## Methods and Materials

### Study Population

**Fig. 1:**
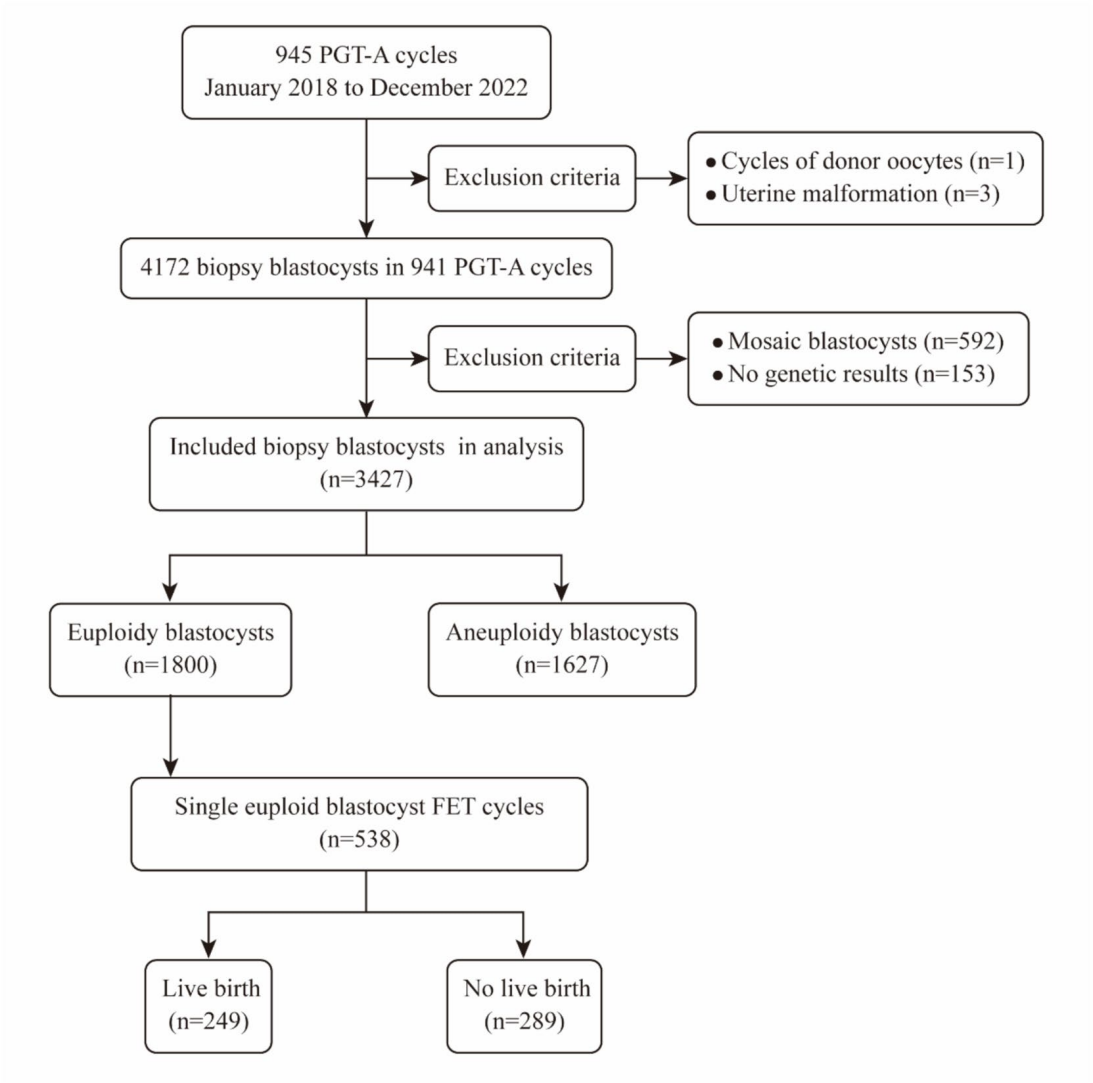
Flow chart of the retrospective cohort study

As shown in Fig. 1, this was a retrospective study conducted at the Reproductive Center of Nanjing Women and Children’s Healthcare Hospital from January 2018 to December 2022. 4172 blastocysts from 941 PGT-A cycles were incorporated, which were fertilized by ICSI and biopsied on day 5/6/7 with next generation sequencing (NGS). 538 blastocysts were selected to transfer according to their NGS results in the following single euploid FET cycles. Cycles were excluded if the embryos were from donor oocytes, biopsy results were inconclusive or involved women were diagnosed with uterine malformation.

### Ovarian Stimulation Protocol

All participants underwent a gonadotropin-releasing hormone (GnRH) antagonist protocol to stimulate the ovarian. Briefly, recombinant follicular stimulating hormone (rFSH, Gonal-F, Merck Serono, Italy) was administrated, varying from 150 to 225 IU per day, on the third day of the menstrual period. The ovarian response and follicular growth were monitored by ultrasound scan and sex hormone levels (FSH, estradiol, luteinizing hormone, and progesterone). Moreover, the dose of gonadotropin (Gn) was adjusted to cause pituitary suppression. When the diameter of at least two dominant follicles reached 18 mm, a dose of 10000 IU human chorionic gonadotrophin (hCG, Lizhu, China) was injected to trigger the ovulation.

### Oocyte Collection, ICSI and Embryo Culture

36 h after hCG injection, vaginal oocyte retrieval was performed under the ultrasound guidance. Then, ICSI was used for all PGT-A cycles in our study, and was conducted 4–6 h after oocyte collection. The embryos were cultured to the blastocyst stage in Vitrolife sequential medium (Goteborg, Sweden) and a humidified atmosphere containing 5% O_2_ and 6% CO_2_ and 89% N_2_.

### Blastocyst Grading, Biopsy and PGT-A

Blastocyst evaluation was assessed based on its degree of expansion and the morphology of ICM and TE prior to embryo biopsy by two experienced embryologists. According to the criteria presented by Gardner and Schoolcraft [1], blastocyst expansion was classified into six grades: 1: the blastocele filling < 50% of blastocyst; 2: the blastocele filling > 50%; 3: a full blastocoele filling the blastocyst; 4: an expanded blastocyst with the thinning zona, the blastocoele volume larger than that of the full blastocyst; 5: a hatching blastocyst, the TE starting to herniate out of the zona; 6: a hatched blastocyst, the blastocyst completely escaping from the zona. The ICM was assigned one of the following three grades: A: numerous tightly packed cells; B: several and loosely packed cells; or C: very few cells. The TE was assigned one of the following three grades: A: many cells organized in epithelium; B: several cells organized in loose epithelium; or C: few large cells. In our center, the eligible blastocytes for biopsy were considered for expansion (4, 5, 6) and ICM or TE grade (AA, AB, AC, BA, BB, BC, CA and CB) on day 5, 6 or 7. TE biopsies were performed as previously described [20]. A laser (Hamilton Thorne Inc., Beverly, USA) was used to open the zona pellucida of blastocyte to allow the passage of biopsy pipette. The biopsy pipette was then pressed against the TE and gently aspirated 4–5 cells into the pipette. 2–3 laser pulses were performed at the junctions among cells and TE cells were separated. Samples were then tested and analyzed by a PGT company (Yikon, Beijing, China) with testing qualification. NGS on the sample of TE cells was conducted under the protocol described by Nagy et al. [21], mainly involving whole-genome amplification (WGA) with a SurePlex DNA Amplification System (Illumina, San Diego, CA, USA), sequencing for 24 chromosome aneuploidy screening with a VeriSeq PGT Kit (Illumina, San Diego, CA, USA) in line with the manufacturer’s recommendations. According to the screening results, blastocysts were diagnosed as euploid, aneuploid or mosaic.

### Vitrification and Warming Procedures

Embryo vitrification and warming followed the previous procedures [6]. After biopsy, blastocysts were firstly equilibrated in vitrification solution 1 (Kitazato BioPharma Co., Shizuoka, Japan) at room temperature for 12 min. Then, the embryos were transferred to vitrification solution 2 at room temperature for 1 min, placed on the tip of Cryotop (Kitazato BioPharma Co.) and put into liquid nitrogen. For thawing, the film strip of the Cryotop device containing the blastocyst was quickly submerged in 0.3 ml of warming thaw solution 1 (Kitazato BioPharma Co.) containing 1.0 M sucrose, at 37℃, for 1 min, followed by transfer of the blastocysts to the second thaw solution 2 containing 0.5 M sucrose and incubation for 3 min. Finally, blastocysts were washed in basic solution 3 and 4 at room temperature for 5 min, and then were transferred into 50 μl droplets of G2 medium (Vitrolife) under mineral oil. Assisted hatching were used before culturing in incubator according to the protocols previously described [22], and blastocysts were transferred after 2–4 h incubation.

### Endometrial Preparation and Embryo Transfer

The choice of endometrial preparation FET was based on clinical discretion and patient preferences. Women with regular menstruation usually underwent natural FET cycles in which follicular growth, endometrial thickness, and type were monitored from day 10 to 12 of their menstrual cycles, using serial transvaginal ultrasonography. When the dominant follicle was larger than 18 mm, the progesterone level ≤ 1.5 ng/ml, and the endometrial thickness ≥ 7 mm (as determined by tri-laminar lining), 10,000 IU hCG was injected. It took about 24–48 h after hCG administration to confirm the ovulation by ultrasound. 10 mg oral dydrogesterone (Abbott Biologicals B.V., Netherlands) was administered three times daily on the day after ovulation to support the luteal phase.

In artificial FET cycles, estradiol valerate (progynova, Bayer, France) was took 4–6 mg per day from the second day of the menstrual cycle for 1 week and adjusted to 8–10 mg on account of the endometrial thickness and serum estradiol (E2) level. This group was further divided according to the addition of GnRH agonist (triptorelin acetate, Diphereline, IPSEN, France), which was administered at the early part of the follicular phase (days 2–4) during the menstrual cycle. After downregulation was confirmed by baseline hormonal (E2 level > 30 pg/ml, luteinizing hormone and FSH levels < 5 IU/L) and transvaginal ultrasound assessment (endometrial thickness < 5 mm), daily 4–6 mg oral estrogen was commenced. When the endometrial thickness reached 7 mm and serum E2 level peaked at 200 pg/ml, 90 mg vaginal progesterone (Crinone, Merck Serono, UK) once a day and 10 mg dydrogesterone three times daily were administered.

It took 6–7 days for embryo transferring after hCG trigger in natural FET cycles and 5 days after progesterone supplementation in artificial FET cycles. One frozen euploid embryo was picked out at a time, based on morphological score and thawed for transferring.

### Outcome Measures

Primary outcomes were euploidy and live birth rates. Euploidy rate was calculated as the number of euploid embryos with 46 chromosomes divided by the total number of biopsied embryos with genetic results. Furthermore, euploidy rate was compared for different blastocyst morphology (expansion, ICM and TE grades) and developmental rate (Day 5, 6 and 7). The primary endpoint was live birth rate after single euploid FET. The live birth rate was defined as the number of live births divided by the sum of embryo transfer cycles included in the cohort. Live birth was considered when a living fetus was born after 28 weeks of pregnancy.

### Statistical Analysis

All data analyses were performed using Stata 9.2 statistical software package (Stata Corp, LP). T test was used to compare the continuous variables; if the variances were far from equal, the Wilcoxon signed rank test was used on maternal age, maternal BMI, duration of infertility, basal FSH, LH, E2 and endometrial thickness. The chi-squared test was used to compare the categorical variables, such as type of infertility, indication for PGT-A and endometrium preparation. Multivariate logistic regression analysis was conducted to confirm whether embryo developmental rate and morphological parameters were independently related to euploidy rate and live birth rate after adjusting for confounding factors. The adjusted variables in the logistic regression model were those with a significance on univariate analysis at *P* < 0.05 or those that could potentially affect euploidy rate and live birth rate, including maternal age, maternal BMI, type of infertility, duration of infertility, indication for PGT-A, endometrium preparation, endometrial thickness, blastocyst developmental rate, expansion, ICM and TE). Odds ratios (ORs) with 95% confidence intervals (CIs) were calculated for euploidy rate and live birth rate in relation to morphology parameters of blastocysts (Expansion, ICM and TE) and developmental rate by multivariate logistic regression analysis. All significance tests were two-tailed, and *P* < 0.05 was considered to be statistically significant.

## Results

### The Relationship of Blastocyst Morphological Parameters and Developmental Rate with Euploidy Rate

A total of 4172 blastocysts from 941 PGT-A cycles were biopsied for ploidy status in this analysis. The mean maternal age of the patients was 34.28 ± 5.08 years. Table 1 displayed the demographic and embryological characteristics of patient in PGT-A cycles. As shown in Table 1, The number and percentage of euploidy and aneuploidy blastocysts were 1800 (43.14%), 1627 (39%). Besides, 592 (14.19%) and 153 (3.67%) blastocysts were determined to be mosaic and no genetic results, respectively; thus, were excluded from this study.

**Table 1:** Demographic and embryological characteristics of patient in PGT-A cycles

Characteristic	
Number of PGT-A cycles (*n*)	941
Maternal age (years)^a^	34.28 ± 5.08
Maternal BMI (kg/m^2^)^a^	22.28 ± 2.85
Duration of infertility (years)^a^	2.63 ± 2.44
Type of infertility, *n* (%)	
Primary	138 (14.67)
Secondary	803 (85.33)
Indication for PGT-A, *n* (%)	
Recurrent pregnancy loss	606 (64.40)
Advanced maternal age	171 (18.17)
Recurrent implantation failure	60 (6.30)
Male factor	104 (11.05)
Basal FSH (mUI/ml)^a^	8.13 ± 2.78
Basal LH (mUI/ml)^a^	4.60 ± 2.25
Basal E2 (pg/ml)^a^	46.53 ± 27.27
Average number of oocytes retrieved^a^	10.24 ± 5.56
Average number of mature oocytes^a^	9.74 ± 5.48
Average number of 2PN cleavages^a^	8.65 ± 5.15
Number of available blastocysts generated(n)	4416
Number of biopsy blastocysts, *n* (%)	4172
Euploid	1800 (43.14)
Aneuploid	1627 (39)
Mosaic	592 (14.19)
No genetic results	153 (3.67)

*PGT-A* preimplantation genetic testing for aneuploidy, *BMI* body mass index, *FSH* follicle-stimulating hormone, *LH* luteinizing hormone, *E2* estradiol

^a^Data are expressed as mean ± SD (standard deviation)

Blastocysts were graded for blastocoele expansion, ICM and TE grades before biopsy, according to the Gardner and Schoolcraft grading system (see Methods). When analyzing the association of these parameters with euploidy rates, we firstly performed a multivariate logistic regression following adjusting for confounding factors (maternal age, maternal BMI, type of infertility, duration of infertility and indication for PGT-A). As shown in Table 2, expansion and TE grades were significantly associated with blastocyst ploidy. Expansion grade 4, as the reference group, had significantly lower euploidy rate than expansion 5 [ORs (95% CI) = 0.69 (0.52–0.92), *P* = 0.011] and 6 [ORs (95% CI) = 0.55 (0.39–0.79), *P* = 0.001]. Similarly, TE grades also appeared striking difference for the euploidy rates between the reference group of grade A and lower quality grades of B [ORs (95% CI) = 1.88 (1.36–2.61), *P* < 0.001] and C [ORs (95% CI) = 4.57 (3.24–6.43), *P* < 0.001]. In addition, blastocysts with grade A ICM demonstrated higher euploidy rates when compared to grade B [ORs (95% CI) = 1.45 (1.00–2.11), *P* = 0.049], representing a light association between ICM and euploidy rate. It was shown that developmental rate had no significant effect on blastocyst euploid rate (day 5 vs day 6, *P* = 0.104; day 5 vs day 7, *P* = 0.204).

**Table 2:** Logistic regression analysis of embryo morphological parameters and developmental rate affecting euploidy rates

		Euploidy (%)	Aneuploid (%)	Crued		Adjusted	
		(*n* = 1800)	(*n* = 1627)	OR (95% CI)	*P* value	OR (95% CI)	*P* value
Expansion^a^	4	50.35 (1507/2993)	49.65 (1486/2993)	1		1	
	5	63.92 (163/255)	36.08 (92/255)	0.57 (0.44–0.75)	< 0.001	0.69 (0.52–0.92)	0.011
	6	72.63 (130/179)	27.37 (49/179)	0.38 (0.27–0.54)	< 0.001	0.55 (0.39–0.79)	0.001
ICM^b^	A	75.69 (137/181)	24.31 (44/181)	1		1	
	B	51.24 (1658/3236)	48.76 (1578/3236)	2.96 (2.1–4.19)	< 0.001	1.45 (1.00–2.11)	0.049
	C	50.00 (5/10)	50.00 (5/10)	3.11 (0.86–11.26)	0.083	1.46 (0.39–5.46)	0.572
TE^c^	A	76.33 (187/245)	23.67 (58/245)	1		1	
	B	62.50 (1095/1752)	37.50 (657/1752)	1.93 (1.42–2.64)	< 0.001	1.88 (1.36–2.61)	< 0.001
	C	36.22 (518/1430)	63.78 (912/1430)	5.68 (4.15–7.77)	< 0.001	4.57 (3.24–6.43)	< 0.001
Developmental rate^d^	D5	61.74 (889/1440)	38.26 (551/1440)	1		1	
	D6	46.74 (881/1885)	53.26 (1004/1885)	1.84 (1.60–2.11)	< 0.001	1.14 (0.97–1.35)	0.104
	D7	29.41 (30/102)	70.59 (72/102)	3.87 (2.50–6.01)	< 0.001	1.36 (0.85–2.18)	0.204

D5, 6, 7, day 5, 6, 7 blastocyst; Expansion, blastocoele expansion, *ICM* inner cell mass, *TE* trophectoderm, *OR* odds ratio, *CI* confidence interval

^a^OR was adjusted for maternal age, maternal BMI, type of infertility, duration of infertility, indication for PGT-A, blastocyst developmental rate, ICM, TE

^b^OR was adjusted for maternal age, maternal BMI, type of infertility, duration of infertility, indication for PGT-A, blastocyst developmental rate, Expansion, TE;

^c^OR was adjusted for maternal age, maternal BMI, type of infertility, duration of infertility, indication for PGT-A, blastocyst developmental rate, Expansion, ICM;

^d^OR was adjusted for maternal age, maternal BMI, type of infertility, duration of infertility, indication for PGT-A, Expansion, ICM, TE

### The Relationship of Blastocyst Morphological Parameters as Well as Developmental Rate with Live Birth Rate

Next, 538 FET cycles of transferred single euploid blastocysts were investigated retrospectively in this study. The demographic characteristics of patients were shown in Table 3, where FET cycles were separated into 2 groups by the pregnancy outcomes. The possibility of live birth was independent of the maternal age (*P* = 0.5936), BMI (*P* = 0.3904), duration of infertility (*P* = 0.3228), basal endocrine levels (FSH: *P* = 0.811; LH: *P* = 0.0892; E2: *P* = 0.5004), and endometrium preparation (*P* = 0.293) and thickness (*P* = 0.2055). However, type of infertility (*P* = 0.012) and indication for PGT-A (*P* = 0.028) were significantly different between the patients with offspring and without offspring.

**Table 3:** Demographic characteristics of patients in frozen-thawed cycles of transferred euploid blastocysts

Characteristic	Live birth	No live birth	*P* value
	(*n* = 249)	(*n* = 289)	
Maternal age (years)^a^	32.35 ± 4.24	32.55 ± 4.36	0.5963
Maternal BMI (kg/m^2^)^a^	22.17 ± 2.83	22.54 ± 3.34	0.3904
Duration of infertility (years)^a^	3.15 ± 2.59	2.94 ± 2.33	0.3228
Type of infertility, *n* (%)			
Primary	53 (21.29)	38 (13.15)	0.012
Secondary	196 (78.71)	251 (86.85)	
Indication for PGT-A, *n* (%)			
Recurrent pregnancy loss	160 (64.26)	205(70.93)	0.028
Advanced maternal age	9 (3.61)	18 (6.23)	
Recurrent implantation failure	19 (7.63)	23 (7.96)	
Male factor	61 (24.50)	43 (14.88)	
Basal FSH (mUI/ml)^a^	7.88 ± 2.56	7.63 ± 2.00	0.811
Basal LH (mUI/ml)^a^	5.22 ± 3.06	4.65 ± 2.22	0.0892
Basal E2 (pg/ml)^a^	44.95 ± 23.30	46.24 ± 21.06	0.5004
Endometrium preparation, *n* (%)			
NC	23 (9.24)	26 (9)	0.293
AC	226 (90.76)	263 (91)	
Endometrial thickness (mm)^a^	8.88 ± 1.62	8.70 ± 1.64	0.2055

*PGT-A* preimplantation genetic testing for aneuploidy, *BMI* body mass index, *FSH* follicle-stimulating hormone, *LH* luteinizing hormone, *E2* estradiol, *NC* natural cycle, *AC* artificial cycle

^a^Data are expressed as mean ± SD (standard deviation)

To explore the effect of embryo morphological parameters and developmental rate on the live birth rates in single euploid FET cycles, a multivariate model, adjusting for maternal age, maternal BMI, type of infertility, duration of infertility, indication for PGT-A, and endometrium preparation and thickness, were analyzed in Table 4. We found that blastocysts with grade A TE had significantly higher live birth rate than those with grade C TE [ORs (95% CI) = 0.45 (0.23–0.90), *P* = 0.024], but no difference from those with grade B TE [ORs (95% CI) = 0.82 (0.45–1.50), *P* = 0.515]. There were no significant relationships between blastocoele expansion (5 vs 4: *P* = 0.451; 6 vs 4: *P* = 0.072), ICM grade (B vs A: *P* = 0.997; C vs A: *P* = 0.819) or developmental rate (D6 vs D5: *P* = 0.356; D7 vs D5: *P* = 0.154) and the rate of live birth, when choosing an euploid blastocyst to transfer in the FET cycles.

**Table 4:** Logistic regression analysis of embryo morphological parameters and developmental rate affecting live birth rate in frozen-thawed cycles of transferred euploid blastocysts

		Live birth (%)	No live birth (%)	Crued		Adjusted	
		(*n* = 249)	(*n* = 289)	OR (95% CI)	*P* value	OR (95% CI)	*P* value
Expansion^a^	4	48.18 (185/384)	51.82 (199/384)	1		1	
	5	43.94 (29/66)	56.06 (37/66)	0.84 (0.5–1.43)	0.525	0.81 (0.47–1.40)	0.451
	6	39.77 (35/88)	60.23 (53/88)	0.71 (0.44–1.14)	0.155	0.64 (0.39–1.04)	0.072
ICM^b^	A	53.66 (22/41)	46.34 (19/41)	1		1	
	B	45.75 (226/494)	54.25 (268/494)	0.73 (0.38–1.38)	0.331	1.00 (0.51–1.96)	0.997
	C	33.33 (1/3)	66.67 (2/3)	0.43 (0.04–5.14)	0.507	0.74 (0.06–9.75)	0.819
TE^c^	A	55.56 (30/54)	44.44 (24/54)	1		1	
	B	50.00 (166/332)	50.00 (166/332)	0.8 (0.45–1.43)	0.449	0.82 (0.45–1.50)	0.515
	C	34.87 (53/152)	65.13 (99/152)	0.43 (0.23–0.81)	0.009	0.45 (0.23–0.90)	0.024
Developmental rate^d^	D5	50.17 (149/297)	49.83 (148/297)	1		1	
	D6	42.13 (99/235)	57.87 (136/235)	0.72 (0.51–1.02)	0.065	0.84 (0.57–1.23)	0.356
	D7	16.67 (1/6)	83.33 (5/6)	0.20 (0.02–1.72)	0.142	0.20 (0.02–1.84)	0.154

D5, 6, 7, day 5, 6, 7 blastocyst; Expansion, blastocoele expansion, *ICM* inner cell mass, *TE* trophectoderm, *OR* odds ratio, *CI* confidence interval

^a^OR was adjusted for maternal age, maternal BMI, type of infertility, duration of infertility, indication for PGT-A, endometrium preparation, endometrial thickness, blastocyst developmental rate, ICM, TE

^b^OR was adjusted for maternal age, maternal BMI, type of infertility, duration of infertility, indication for PGT-A, endometrium preparation, endometrial thickness, blastocyst developmental rate, Expansion, TE;

^c^OR was adjusted for maternal age, maternal BMI, type of infertility, duration of infertility, indication for PGT-A, endometrium preparation, endometrial thickness, blastocyst developmental rate, Expansion, ICM;

^d^OR ratio was adjusted for maternal age, maternal BMI, type of infertility, duration of infertility, indication for PGT-A, endometrium preparation, endometrial thickness, Expansion, ICM, TE

## Discussion

In conclusion, our retrospective study described the correlations between blastocyst developmental rate, morphologic grades and euploidy rates in PGT-A cycles, and the influence of developmental rate, morphologic grades on live birth rates in the FET cycles of euploid blastocysts.

We found that blastocyst expansion and TE grade had the strongest association with the euploidy rate. There were only weakly significant differences in euploidy rate among blastocysts with grade B ICM, compared to those with grade A ICM. Meanwhile, our model confirmed that assessment of euploidy blastocysts through TE grade could offer useful guidance for selecting blastocysts with high chance of live birth in FETs. However, neither the euploidy rate nor live birth rate was associated with developmental rate.

Some studies concluded that euploid blastocysts took a higher probability of acquiring excellent morphology than aneuploid blastocysts, as reflected in the top grades of ICM and TE, higher grade of expansion [23, 24]. Another analysis by Johnson et al. [25] described shorter time to blastulation, expansion and hatching in euploid blastocysts. In our study, the morphologic grade of TE had the strongest association with the chromosome ploidy and live birth, which offered a useful guidance for selecting an euploidy blastocyst with the highest chance of acquiring a progeny in FET cycles. TE grades were more closely related to blastocyst euploidy than ICM grades in previous studies [14, 26]. Alfarawati et al. [16] reported that declining TE grade was correlated with increasing aneuploidy rate. The above phenomenon is properly attributed to the fact that TE differentiates into the placenta, facilitating the communication between the embryo and the mother after implantation, which in turn determines the development of the embryo [27, 28]. Meanwhile, blastocoele expansion serves as one of the most crucial morphological indicators for predicting successful live births following the single blastocyst implant [29]. However, we found the declined pertinence of expansion, compared to TE grades, mainly in the sense that the pregnancy outcome of ploidy blastocysts was independent of the size of blastocysts, despite the strong association between blastocyst ploidy and dilatation.

On the other hand, the quality of ICM has also been demonstrated to be associated with euploidy or live birth rates in some researches [30–32], while there was weakly significant difference in euploidy rates and live birth rates between grade A and B ICM in the study. These findings were consistent with the conclusions of Anderson R et al., who considered that once a euploid embryo was transferred, live birth success was attained independent of embryo quality [33]. The cells used for euploidy detection are derived from TE biopsy, which may explain the lack of correlation between ICM grades and NGS results. Similarly, our study indicated that neither the euploidy rates nor live birth rates were associated with developmental rates. In fact, the relationship between blastocyst development rates and euploidy rates or live birth rates are difficult to reconcile. Some studies confirmed that blastocysts on day 5 had higher euploidy rate than that on day 6 [13, 34], and the lowest euploidy rate was observed in day 7 blastocysts [35]. In FET cycles, the live birth rate of day 5 euploid embryos was obviously higher, compared with that of day 6 embryos [36]. In contrast, other retrospective studies showed a similar aneuploidy rate in faster and slower blastocysts [16, 37], and the implantation rate of euploid embryos at day 7 were not different from those at day 5 or 6 [19]. On a cautionary note, selection bias should be taken into account, as blastocysts with grade C ICM or on day 7 are rarely chosen for PGT-A test or transfer in this study.

In sum, our study had several key strengths. Firstly, the samples collected from a single center ensured the uniformity of treatment and laboratory procedures throughout the entire study duration. Secondly, we employed multivariate logistic regression models, adjusting for potential confounders, to validate our study findings. At the same time, some limitations of this study are noted, mainly including the inherent limitations of the retrospective cohort study and selection bias due to the optimization of good-quality blastocysts for transfer in view of patient requirements and the purpose of ART. Therefore, more prospective studies with large samples are needed to verify our findings.

## Conclusion

To sum up, morphologically good embryos, including those with a high degree of expansion, ICM and TE grades, were found to have a higher probability of euploidy than poor-quality embryos in the PGT-A cycles. In addition, TE quality requires particular attention in the single euploid blastocyst FET cycles, since TE grades were associated with the probability of live birth.

## Data Availability

The data that support the findings of this study are available from the corresponding author upon reasonable request.
